# Prognostic Nutritional Index Predicts Toxicity in Head and Neck Cancer Patients Treated with Definitive Radiotherapy in Association with Chemotherapy

**DOI:** 10.3390/nu13041277

**Published:** 2021-04-13

**Authors:** Giuseppe Fanetti, Jerry Polesel, Elisabetta Fratta, Elena Muraro, Valentina Lupato, Salvatore Alfieri, Carlo Gobitti, Emilio Minatel, Fabio Matrone, Angela Caroli, Alberto Revelant, Marco Lionello, Viviana Zammattio Polentin, Andrea Ferretti, Roberto Guerrieri, Paola Chiovati, Andy Bertolin, Vittorio Giacomarra, Antonino De Paoli, Emanuela Vaccher, Giovanna Sartor, Agostino Steffan, Giovanni Franchin

**Affiliations:** 1 Division of Radiotherapy, Centro di Riferimento Oncologico di Aviano (CRO) IRCCS, 33081 Aviano, Italy; cgobitti@cro.it (C.G.); eminatel@cro.it (E.M.); fabio.matrone@cro.it (F.M.); angela.caroli@cro.it (A.C.); alberto.revelant@cro.it (A.R.); viviana.zammattio@cro.it (V.Z.P.); aferretti@cro.it (A.F.); adepaoli@cro.it (A.D.P.); gfranchin@cro.it (G.F.); 2 Unit of Cancer Epidemiology, Centro di Riferimento Oncologico di Aviano (CRO) IRCCS, 33081 Aviano, Italy; polesel@cro.it; 3 Division of Immunopathology and Cancer Biomarkers, Centro di Riferimento Oncologico di Aviano (CRO) IRCCS, 33081 Aviano, Italy; efratta@cro.it (E.F.); emuraro@cro.it (E.M.); roberto.guerrieri@cro.it (R.G.); asteffan@cro.it (A.S.); 4 Division of Otolaryngology, “Santa Maria degli Angeli” General Hospital, 33170 Pordenone (PN), Italy; vittorio.giacomarra@asfo.sanita.fvg.it; 5 Division of Medical Oncology and Immune-Related Tumors, Centro di Riferimento Oncologico di Aviano (CRO) IRCCS, 33081 Aviano, Italy; salvatore.alfieri@cro.it (S.A.); evaccher@cro.it (E.V.); 6 Division of Otolaryngology, Vittorio Veneto General Hospital, ULSS2 Marca Trevigiana, 31029 Vittorio Veneto, Italy; marco.lionello@aulss2.veneto.it (M.L.); andy.bertolin@aulss2.veneto.it (A.B.); 7 Division of Medical Physics, Centro di Riferimento Oncologico di Aviano (CRO) IRCCS, 33081 Aviano, Italy; pchiovati@cro.it (P.C.); gsartor@cro.it (G.S.)

**Keywords:** prognostic nutritional index (PNI), head and neck cancer, weight loss, mucositis, radiotherapy

## Abstract

Background: The Prognostic Nutritional Index (PNI) is a parameter of nutritional and inflammation status related to toxicity in cancer treatment. Since data for head and neck cancer are scanty, this study aims to investigate the association between PNI and acute and late toxicity for this malignancy. Methods: A retrospective cohort of 179 head and neck cancer patients treated with definitive radiotherapy with induction/concurrent chemotherapy was followed-up (median follow-up: 38 months) for toxicity and vital status between 2010 and 2017. PNI was calculated according to Onodera formula and low/high PNI levels were defined according to median value. Odds ratio (OR) for acute toxicity were calculated through logistic regression model; hazard ratios (HR) for late toxicity and survival were calculated through the Cox proportional hazards model. Results: median PNI was 50.0 (interquartile range: 45.5–53.5). Low PNI was associated with higher risk of weight loss > 10% during treatment (OR = 4.84, 95% CI: 1.73–13.53 for PNI < 50 versus PNI ≥ 50), which was in turn significantly associated with worse overall survival, and higher risk of late mucositis (HR = 1.84; 95% CI:1.09–3.12). PNI predicts acute weight loss >10% and late mucositis. Conclusions: PNI could help clinicians to identify patients undergoing radiotherapy who are at high risk of acute and late toxicity.

## 1. Introduction

Definitive radiotherapy in association with induction and/or concurrent chemotherapy represents a non-surgical treatment choice for patients with head and neck cancer (HNC) [1]. Although these strategies improve the oncologic outcomes, HNC patients are exposed to a broad range of complications, which deserve continuous evaluation and specific management. Radiation induced toxicity has a significant impact on the HNC patients’ quality of life and may lead to treatment discontinuation. In particular, radiotherapy induces mucositis and dysphagia as well as xerostomia and dysgeusia, which ultimately cause malnutrition and weight loss [2]. Furthermore, malnutrition is already present at diagnosis in 30–50% of HNC patients, and it may worsen during treatment [3]. Notably, a long history of tobacco and/or alcohol exposure further correlates with a poor nutritional status in these patients [4].

The majority of research is focused on prognostic factors helping clinicians to identify patients at higher risk of recurrence and death from HNC. So far, only TNM stage, HPV status and patients related features (i.e., smoking/alcohol consumption, performance status, age) have been recognized as prognostic factors for HNC [5,6]. Several efforts have recently been made worldwide in the field of genetics, epigenetics, and OMICs (radiomics, metabolomics, dosiomics) in order to identify new parameters that may improve risk stratification of HNC patients and calibrate treatment effects [7,8,9,10]. On the contrary, predictive factors of severe treatment related toxicity and above all radiation induced toxicity are lacking and mostly refer to dosimetric parameters strictly connected to treatment planning in radiotherapy [11]. Additionally, models have been proposed to identify the probability of normal tissues complications (NTCP) to evaluate radiation dose related risk of adverse events [12]. In the recent years, also data from radiomics and radiologic imaging analysis increased the knowledge in early detection of patients at risk of radiation related toxicity [13,14]. Those biomarkers, although promising, need further validation and require dedicated specialists and resources, thus limiting the application in nontertiary or academic hospitals.

Interestingly, systemic inflammation has been studied as a marker of poor outcome and can be easily evaluated through peripheral blood tests. Several parameters have been recognized as predictive factors in HNC [15,16], such as lymphopenia, high neutrophil-to-lymphocyte ratio or platelet-to-lymphocyte ratio [17,18]. Among the biomarkers of systemic inflammation, the prognostic nutritional index (PNI) is calculated by combining the circulating albumin concentration with the lymphocyte count. Therefore, PNI has also been recognized as an accurate parameter reflecting nutritional and inflammation status [19]. In the last few years, PNI has been extensively investigated as predictor of poor outcome in several types of cancer [20,21,22,23,24,25,26,27,28,29,30], including HNC [31,32,33,34]. Although a number of studies have evaluated PNI in relation to treatment toxicity [35,36,37,38], only few ones focused on HNC [39,40,41,42].

On these grounds, our study aimed at investigating the predictive role of PNI for severe acute and late radiation induced toxicity in patients with HNC treated with radiotherapy in association with induction and/or concurrent chemotherapy. Moreover, we also evaluated the impact of PNI on survival in terms of disease-free survival (DFS) and overall survival (OS).

## 2. Materials and Methods

### 2.1. Patients

The present retrospective study included HNC patients consecutively treated with definitive radiotherapy in association with induction and/or concomitant chemotherapy at our Institution, between January 2010 and December 2017. Patients were included in this analysis if they met the following inclusion criteria: (a) age ≥ 18 years; (b) histological diagnosis of cancer of the rhinopharynx, oropharynx, hypopharynx or larynx; (c) therapeutic indication for definitive radiotherapy in association with chemotherapy with intensity modulated radiotherapy (IMRT); (d) radical radiation dose delivered with conventional fractionation; (e) assessment of toxicity during and at the end the IMRT; f) 6-month minimum follow-up after treatment completion; (g) available baseline assessment of serum albumin, lymphocyte count, height and weight. Patients treated with palliative or adjuvant intent were excluded as well as patients with distant metastases or those receiving radical radiotherapy alone (without sequential or concurrent chemotherapy).

### 2.2. Treatment Schedule

At baseline, all patients were staged according to TNM, 7th edition, and they were evaluated for treatment indication by a multidisciplinary tumor board, including at least the ear-nose-throat surgeon, the radiotherapist and the oncologist, fully focused on the management of HNC patients. A total dose of 70.95 Gray (Gy) in 33 fractions was delivered to the Planning Target Volume (PTV) of the macroscopic disease. A total dose of 62.70 Gy in 33 fractions was delivered to the PTV high risk and 56.10 Gy in 33 fractions to the PTV low risk. The association with chemotherapy was define on the basis of TNM stage and general conditions of patients, in agreement with institutional policy and international guidelines. Concomitant cisplatin (80–100 mg/m^2^) was administered at day 1-22-43 of radiotherapy to patients with T1–T3 and N0–N1 stage. For patients with T4 and/or N2–N3 stage, induction chemotherapy was preferred and consisted of 3 cycles of cisplatin (75 mg/m^2^) and docetaxel (75 mg/m^2^) on day 1 and 5-fluorouracil (750 mg/m^2^/d) on days 1–5 (TPF scheme) repeated every 21 days. After induction chemotherapy, concurrent weekly cisplatin (40 mg/m^2^) was administered concomitantly with radiotherapy.

### 2.3. Response and Toxicity Assessment

Acute toxicity (i.e., weight loss, mucositis, dermatitis) was evaluated every week during IMRT with clinical examination and fiberoptic laryngoscopy both performed by the radiation oncologist; maximum acute toxicity during IMRT was considered for the present analysis. Patients were evaluated for treatment outcome at the end of IMRT. Thereafter, patients were followed-up every three months after the end of IMRT for the first two years, then every 4–6 months until the 5th year after IMRT, in agreement with internal procedures and national and international guidelines. During each follow-up visit, treatment response was assessed according to response evaluation criteria in solid tumors (RECIST) through physical examination, fibrolaryngoscopy, and radiologic imaging. Late toxicities (i.e., xerostomia, dysphagia, dysgeusia, mucositis, hypothyroidism, hearing loss) were also evaluated during each follow-up examination.

All toxicities were scored according to Common Terminology Criteria for Adverse Events (CTCAE) scale version 4.03. Severe acute toxicity was defined as grade ≥3; severe late toxicity was defined as grade ≥2 (i.e., impacting on patients’ quality of life). Weight loss was defined as significant if the decrease was >10% compared to baseline; hypothyroidism, hearing loss were classified as presence/absence.

### 2.4. Clinical and Nutritional

At baseline, the following parameters were collected: gender, age, Karnofsky Performance Status (KPS), Charlson’s Comorbidity Index (CCI), Body Mass Index (BMI), lymphocyte count, serum albumin, drinking and smoking habits, site of disease and TMN stage. Drinkers were defined as patients who had regularly drunk more than one glass/day of alcoholic beverages (e.g., wine, beer or spirit); smokers were patients who smoked regularly more than one cigarette/day. Pre-treatment PNI was calculated according to the Onodera formula [43] as 10(serum albumin) + 0.005·(lymphocyte count).

### 2.5. Statistical Analysis

Differences in clinical and nutritional parameters among patients with and without toxicities were evaluated by Wilcoxon test non-parametric test for quantitative variables or by Fisher exact test for qualitative variables. Each nutritional parameter, low and high levels were defined according to median value.

The association between nutritional parameter and acute toxicity was evaluated through logistic regression model, accounting for potential confounders (i.e., gender, age, cancer site, performance status, body mass index, stage and ever smoking).

Time to event was calculated from the date of the end of therapy to the event of interest or death, whichever occurred first (median follow up: 38 months; interquartile range: 21–67 months). Events of interest were death for overall survival (OS), disease recurrence or death for disease-free survival (DFS), and clinical appearance of toxicity for toxicity-free survival. The impact of nutritional parameters on clinical outcomes was analyzed with non-parametric Kaplan–Meier method. Curves were stratified according to the categories of PNI and differences evaluated with log-rank test [44]. To account for potential confounding factors, the association of PNI with clinical outcomes was further evaluated with the Cox proportional hazards model, adjusting for gender, age, cancer site, performance status, body mass index, stage and ever smoking. Statistical significance was claimed for *p* < 0.05 (two-sided).

## 3. Results

Table 1 shows PNI levels according to potential confounders of the association between PNI and oncological outcomes. The majority of patients were men (70.9%) and patients aged ≥ 55 years (64.2%); no patients reported BMI < 18.5 kg/m^2^ at diagnosis. The majority of patients reported TNM stage IV (60.9%), and those with stage II were all diagnosed with nasopharyngeal cancer with positive nodes. Median PNI was 50.0 (interquartile range: 45.5–53.5), and it was higher in ever smokers and in patients with 0–2 Charlson’s Comorbidity Index.

**Figure 1:**
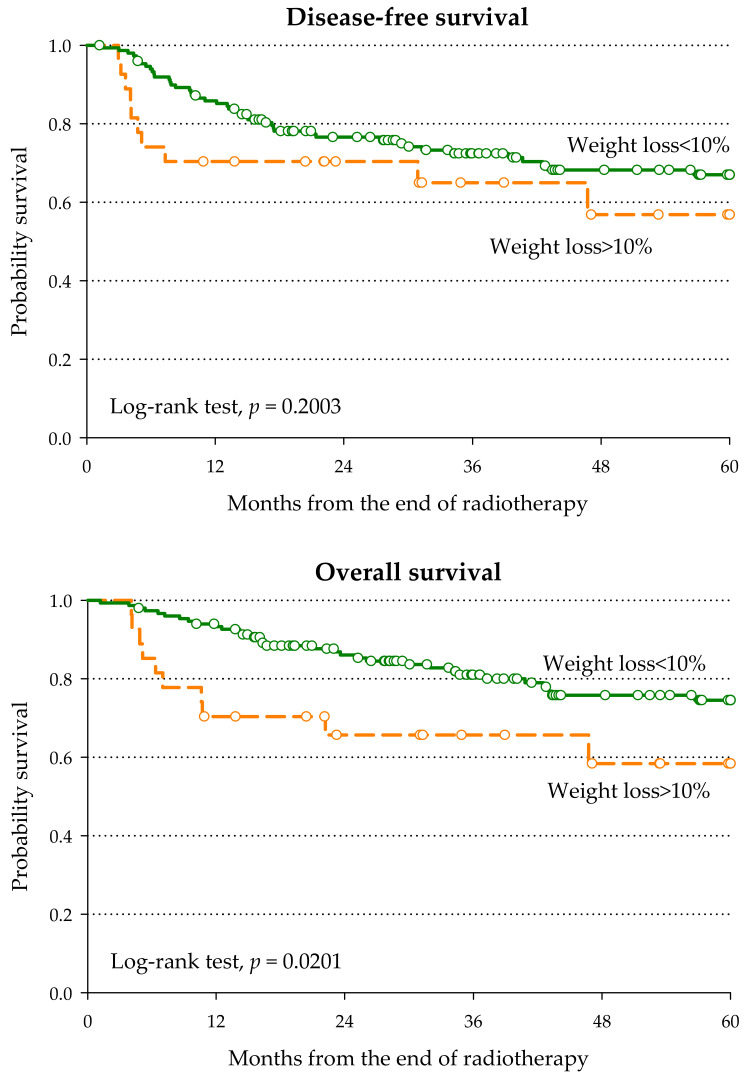
Disease-free survival and overall survival according to weight loss during radiotherapy.

Overall, 120 patients (67.0%) experienced severe acute G3–G4 toxicity (Table 2), and the three most frequent acute toxicities were mucositis (*n* = 84, 46.9%), weight loss > 10% (*n* = 27, 15.1%), and dermatitis (*n* = 26, 14.5%). PNI <5 0 was significantly associated with higher risk of weight loss during treatment (OR = 4.84, 95% CI: 1.73–13.53), and it seemed mainly due to low albumin level (OR = 2.96; 95% CI: 1.16–7.57). This association is of clinical relevance, since patients who experienced weight loss > 10% during treatment reported worse survival than those who maintained their weight (5-year OS: 58.4% versus 74.5%; *p* = 0.0201—Figure 1), with a multivariable HR of death of 2.44 (95% CI: 1.15–5.15).

Overall, 120 patients (67.0%) reported at least one late toxicity (Table 3): xerostomia (*n* = 43, 24.0%), hypoacusia (*n* = 32, 17.9%), and hypothyroidism (*n* = 25, 14.0%) were the most frequent ones. PNI < 50 was significantly associated with a higher risk of mucositis (HR = 1.84; 95% CI: 1.09–3.12); the occurrence of late toxicities was not significantly associated with PNI, albumin or lymphocytes. Patients with low PNI showed a worse, though not significant, DFS (HR = 1.56; 95% CI: 0.92–2.66) and OS (HR = 1.57; 95% CI: 0.83–2.97) than those with high PNI.

## 4. Discussion

The present study showed that a low PNI level was significantly associated with important weight loss during radiotherapy in association with induction and/or concurrent chemotherapy for HNC, which was in turn associated with worse survival outcomes. Further, patients with a low PNI had a higher risk of developing late mucositis.

Weight loss has been widely recognized as a predictor of poor outcome in several neoplasms, and it is of great concern especially in HNC. Several factors cause weight loss such as pre-treatment nutritional deficiencies, acute mucositis, tobacco smoking, radiation doses to organs at risk (such as parotids), serum albumin levels, BMI [6,45,46,47,48]. In our study, 15.1% of patients experienced a weight loss >10% during treatment, and for these patients, OS decreased significantly.

The peculiarity of PNI is to be a marker of both systemic inflammation and nutritional status. Indeed, it derives from serum albumin and lymphocyte count, which are widely recognized markers of nutritional status and inflammation. Serum albumin levels decrease in chronic disease and in cancer as well. Mechanism inducing hypoalbuminemia are both exogenous (i.e., the decrease in the intake of proteins and calories) and endogenous (i.e., the activation of systemic inflammation that favors catabolism) [49]. Both pathways are possible in patients with HNC. Despite the fact that hypoalbuminemia is frequently considered an important biochemical marker of nutritional status because of its association with malnutrition [50], albumin lacks specificity as it may decrease as a consequence of other comorbidities [51]. In our study population, however, patients with 3–7 Charlson’s Comorbidity Index reported an albumin level (median = 4.1 g/dL; Q1–Q3: 3.8–4.4 g/dL) similar to that reported in those with 0–2 Charlson’s Comorbidity Index (median = 4.0 g/dL; Q1–Q3: 3.5–4.4 g/dL). Lymphocytes belong to the adaptive immune system, and tumor infiltrating lymphocytes play a crucial role in the immune response against cancer. Lymphopenia has been associated with poor outcome [52]. Although serum albumin and lymphocyte count may both have drawbacks as markers of inflammation and nutritional status, it should be noted that their combination into the PNI lead to a more powerful multidimensional index for the identification of patients at risk of toxicity.

Higher levels of PNI have been associated with favorable prognosis in lung [24,25], colorectal [30], breast [26], prostate [29], and cervical [21] cancers. Nonetheless, few reports have investigated the role of PNI in HNC, so far. In most of the published studies, higher pre-treatment PNI was associated with a better outcome and OS in HNC patients who underwent surgery [31,32,53,54] as well as in those who were treated with definitive radiotherapy [33,34]. In our study, we included only HNC patients treated with definitive radiotherapy with sequential or concurrent chemotherapy and, our results showed a possible role on DFS and OS prediction, in line with previously published data.

A low PNI has recently been recognized to predict iatrogenic toxicity and complication in oncologic patients undergoing surgery or chemotherapy [38,55]. Low PNI has been associated with severe acute adverse events (i.e., toxicity of grade ≥ 3) of any type in HNC patients treated with radical or adjuvant radiotherapy [39], without distinction of single toxicity type. Recently, Chang and colleagues [56] have reported higher rates of feeding tube placement, G3–G4 hematological toxicities, and sepsis during chemoradiotherapy in HNC patients with low PNI. In our cohort, an association emerged between PNI and acute weight loss during radiotherapy in association with induction and/or concurrent chemotherapy. The risk of feeding tube placement has not been evaluated in the present study since only one case of feeding tube placement due to severe dysphagia was reported. On the contrary, in our study patients with higher PNI reported a major risk of acute mucositis. This finding appears in contradiction with the published literature, and it is difficult to interpret. However, this observation could suggest that weight loss in patients with lower PNI may be due to a mechanism unrelated to mucositis.

The published literature refers mainly to the association of PNI with the onset of acute surgical or radiation induced complications. We evaluated also the association of PNI with late radiation related toxicity. In our report, patients with higher levels of PNI had 2-fold lower risk to develop chronic mucositis.

Although the above reported literature mainly focused on patients living in the far East (i.e., China and Japan), the interest for PNI in HNC has recently emerged in Europe as well. For instance, Bruixola and colleagues [57] evaluated a cohort of 145 HNC patients treated with induction chemotherapy followed by chemoradiation delivered with a 3D conformal technique and, consistently with our data, they found that low PNI levels correlated with worse survival.

The results from this study may help to reach beyond the limits of PNI application in clinical practice. Firstly, a cut-off value to stratify patients is still lacking with investigations on the topic suggesting values ranging from 40 to 52 [31,32,33,34,39], and our cut-off was in line with current literature. Secondly, radiation technique for HNC is in continuous evolution towards advanced treatment modalities that are associated with low toxicity. For this purpose, our study was restricted to patients treated with IMRT, a technique that minimizes organs at risk (OARs) exposure to radiotherapy ensuing low toxicity.

Further, a detailed analysis of the impact of PNI on different kinds of toxicity was provided in contrast with other studies focusing only on few toxicities or on high grade toxicity, in general. Finally, our analysis was directed to both outcome and toxicity risk assessment in the same population.

Some study limitations have to be acknowledged. Firstly, the retrospective nature of this study may have introduced selection bias as patients’ eligibility was driven by the availability of serum albumin level before treatment. Furthermore, as PNI calculation is based on a single blood sampling, it may be affected by causal perturbation. However, both serum albumin [51] and lymphocytes count [58] have been reported to be quite stable over time, in terms of few weeks, and their daily variation is unlike to substantially change PNI level. In addition, this study population included different cancer sub-sites, so that the study findings may depend on cancer mix. Therefore, the value of PNI needs to be prospectively validated in larger cohort studies, where subsite-specific analyses could be performed. Another limitation consisted in the lack of patient-reported outcomes. Notably, self-reported patients’ assessment of toxicities and quality of life through validated questionnaires is rapidly emerging in medical oncology [57]. This can represent another important perspective that needs to be combined with the physicians’ evaluation when measuring the impact of the toxicity burden from cancer treatments. Furthermore, the continuous presence of a specialist in clinical nutrition in the multidisciplinary board would have improved the management of weight loss during radiotherapy. The mono-institutional nature of this investigation could also represent another study limitation, as well as the variability of combinations of chemotherapy regimen and doses with radiotherapy. In addition, the heterogeneity of treatment schedules could have impacted on the association between PNI and survival outcomes. On the other hand, in our study, patients were evaluated for treatment by the same multidisciplinary tumor board, delivering treatment according to the same standard procedures.

## 5. Conclusions

The present findings confirmed the usefulness of PNI in daily practice, which combines serum albumin and lymphocyte count into a powerful multidimensional indicator of both nutritional and immunological status. In contrast to serum albumin and lymphocyte counts taken separately, PNI allows the identification of patients at risk of toxicity. PNI is easily obtainable from a routine blood test with limited cost, thus resulting in high sustainability for the healthcare systems. Furthermore, PNI identifies patients at higher risk of severe sequelae after radiotherapy in association with induction and/or concurrent chemotherapy, such as weight loss that strongly impacts not only on patients’ quality of life but also survival. PNI could help clinicians in taking some actions (such as improving nutritional counseling) as well as reducing the dose to OARs (i.e., parotid glands, oral cavity) at major risk of late complications or administering drugs to improve OARs function [59]. These considerations could be discussed with patients in a perspective of informed tailored therapeutic programs.

## Figures and Tables

**Table 1:** Prognostic Nutritional Index according to socio-demographic and clinical characteristics.

Characteristics	*n*	(%)	Prognostic Nutritional Index		Kruskal–Wallis Test
			Median	(Q1–Q3)	
Overall	179		50.0	(45.5–53.5)	
Sex					
Man	127	(70.9)	49.5	(45.5–53.5)	*p* = 0.782
Woman	52	(29.1)	50.0	(45.3–54.1)	
Age (years)					
<55	64	(35.8)	49.5	(45.5–53.8)	*p* = 0.093
55–64	52	(29.1)	51.0	(47.5–54.9)	
≥65	63	(35.2)	49.5	(43.0–53.0)	
Smoking habits					
Never	49	(27.4)	48.0	(43.5–52.0)	*p* = 0.022
Ever	130	(72.6)	50.6	(46.0–54.5)	
Drinking habits					
Never	87	(48.6)	50.0	(45.5–53.5)	*p* = 0.922
Ever	92	(51.4)	49.5	(45.5–53.9)	
Body Mass Index (kg/m^2^)					
18.5–25	86	(48.0)	49.3	(45.0–54.0)	*p* = 0.595
25-34	64	(35.8)	50.0	(46.3–54.4)	
≥35	29	(16.2)	49.0	(45.5–52.5)	
Performance status (ECOG)					
0	28	(15.6)	48.9	(46.5–54.0)	y = 0.693
1–2	151	(84.4)	50.0	(45.0–53.5)	
Charlson’s Comorbidity Index					
0–2	117	(65.4)	50.0	(47.0–53.8)	*p* = 0.039
3–7	62	(34.6)	48.8	(43.0–53.5)	
Cancer site					
Rhinopharynx	51	(28.5)	48.0	(44.0–53.0)	*p* = 0.083
Oropharynx	86	(48.0)	50.0	(46.0–53.4)	
Hypopharynx/Larynx	42	(22.5)	52.0	(45.5–55.3)	
TNM stage					
II	24	(13.4)	48.4	(46.3–55.5)	*p* = 0.750
III	46	(25.6)	50.3	(45.5–54.0)	
IV	109	(60.9)	49.5	(45.5–53.0)	
Chemotherapy regimen					
Sequential	141	(78.8)	50.0	(46.0–54.4)	*p* = 0.130
Concurrent	38	(21.2)	48.3	(44.9–52.0)	

**Table 2:** Odds ratio (OR) and 95% confidence interval (CI) ^1^ for acute G3–G4 toxicity according to nutritional factors.

Acute Toxicity	*n*	PNI		Albumin (g/dL)		Lymphocytes/mm^3^	
		≥50	<50	≥4.1	<4.1	≥1780	<1780
		Ref.	OR (95% CI)	Ref.	OR (95% CI)	Ref.	OR (95% CI)
All	120	1	1.17 (0.61–2.25)	1	1.12 (0.58–2.16)	1	1.00 (0.51–1.98)
Mucositis	84	1	0.70 (0.38–1.31)	1	0.72 (0.39–1.35)	1	1.00 (0.53–1.90)
Weight loss	27	1	4.84 (1.73–13.53)	1	2.96 (1.16–7.57)	1	1.53 (0.61–3.85)
Dermatitis	26	1	1.77 (0.72–4.34)	1	1.62 (0.66–3.94)	1	1.04 (0.42–2.57)
Dysgeusia	19	1	1.05 (0.34–3.26)	1	1.13 (0.36–3.53)	1	0.59 (0.18–2.00)

^1^ Estimated through logistic regression model, adjusting for gender, age, cancer site, performance status, tobacco smoking, cancer stage, and BMI >25 kg/m^2^. PNI: Prognostic Nutritional Index.

**Table 3:** Hazard ratio (HR) and 95% confidence interval (CI) ^1^ for late G2–G4 toxicity, disease-free survival (DFS) and overall survival (OS), according to nutritional factors.

Outcome	*n*	PNI		Albumin (g/dL)		Lymphocytes/mm^3^	
		≥50	<50	≥4.1	<4.1	≥1780	<1780
		Ref.	OR (95% CI)	Ref.	OR (95% CI)	Ref.	OR (95% CI)
**Late toxicity**							
All	120	1	1.05 (0.75–1.48)	1	1.09 (0.77–1.55)	1	1.10 (0.78–1.55)
Xerostomia	43	1	1.02 (0.64–1.65)	1	1.09 (0.68–1.76)	1	0.98 (0.61–1.57)
Hypoacusia	32	1	1.22 (0.76–1.94)	1	1.40 (0.87–2.24)	1	0.96 (0.60–1.54)
Hypothyroidism	25	1	1.43 (0.88–2.34)	1	1.32 (0.80–2.19)	1	1.09 (0.67–1.77)
Dysgeusia	21	1	1.33 (0.80–2.20)	1	0.86 (0.51–1.44)	1	1.11 (0.67–1.84)
Mucositis	15	1	1.84 (1.09–3.12)	1	1.29 (0.76–2.18)	1	1.08 (0.64–1.83)
**Survival**							
DFS	62	1	1.56 (0.92–2.66)	1	1.30 (0.76–2.23)	1	1.00 (0.59–1.69)
OS	51	1	1.57 (0.83–2.97)	1	1.11 (0.60–2.02)	1	1.01 (0.61–1.97)

^1^ Estimated through Cox proportional hazards model, adjusting for gender, age, cancer site, performance status, tobacco smoking, cancer stage, and BMI >25 kg/m^2^. PNI: Prognostic Nutritional Index.

## Data Availability

The data are available to the corresponding authors (G.F. (Giuseppe Fanetti) and V.L.) upon reasonable request.
